# Quality in perinatal care: applying performance measurement using joint commission on accreditation of healthcare organizations indicators in Italy

**DOI:** 10.1186/s12874-019-0722-z

**Published:** 2019-04-24

**Authors:** Claudia Pileggi, Lorena Squillace, Mariavalentina Giordano, Rosa Papadopoli, Aida Bianco, Maria Pavia

**Affiliations:** Department of Health Sciences, University of Catanzaro “Magna Græcia”, Via Tommaso Campanella, 88100 Catanzaro, Italy

**Keywords:** Perinatal care, Quality measure, Hospital performance

## Abstract

**Background:**

Maternal and child health are internationally considered to be among the best measures for assessing health-care quality. The study was carried out with the following aims: 1) to assess the quality of perinatal care (PC) by measuring the frequencies of the five PC indicators developed by the Joint Commission on Accreditation of Healthcare Organizations (JCAHO) and comparing results with international standards; 2) to examine whether maternal, pregnancy care and neonatal characteristics could be factors associated with the quality of perinatal care hospital performance, measured through these indicators.

**Methods:**

We retrospectively reviewed medical charts of women over the age of 18 who experienced delivery in Gynecology/obstetrics wards between January–December 2016, and those of their newborns hospitalized in the Neonatology or Neonatal Intensive Care Unit (NICU) of a public non-teaching hospital in Catanzaro (Italy). Indicators were calculated according to the methodology specified in the manual for JCAHO measures. Univariate and multivariate analyses were performed to test the independent association of maternal, pregnancy care and neonatal characteristics on the adherence to JCAHO PC indicators.

**Results:**

The records of 1943 women and 1974 newborns were identified and reviewed in order to be included in at least one of the PC indicators. Elective/early-term delivery, was performed in 27.6% of eligible women, far from the recommended goal (0%); cesarean section in nulliparous women with a term, singleton baby in a vertex position exceeded the suggested target of < 24% and the adherence to antenatal steroids administration was suboptimal (87%). Results of the exclusive breastfeeding indicator achieved a better performance (81%) and compliance with the PC-04 indicator was satisfactory with only 0.4% healthcare-associated bloodstream infection developed in eligible newborns.

**Conclusions:**

This is the first study performed in Italy that has evaluated the quality of PC by using all the five JCAHO indicators. The application of this feasible set of indicators allowed us to measure several aspects of PC for which there is no standardized monitoring system in Italy. Our findings revealed significant deficiencies in the adherence to recommended processes of PC and suggest that there is still substantial work required to improve care.

**Electronic supplementary material:**

The online version of this article (10.1186/s12874-019-0722-z) contains supplementary material, which is available to authorized users.

## Introduction

Maternal and child health is a public health priority, because pregnancy, childbirth and puerperium are leading causes of hospitalization for women, and birth-related events are internationally considered to be among the best measures for assessing health-care quality. The Joint Commission on Accreditation of Healthcare Organizations (JCAHO) developed the Perinatal Care (PC) core measure set that includes five metrics with sufficient evidence that better performance are clinically important and are possible with system and process improvement [1, 2]. These core measures were chosen from a broader set among those recommended by the National Quality Forum (NQF) by a technical advisory panel of experts in perinatal care. The benefit of the core measures is that they provide a national, standardized set of quality metrics that hospitals can use [3].

In Italy, since 2010, the Outcomes National Plan (Piano Nazionale Esiti - PNE), started up by the National Agency for Regional Health Services (Agenzia Nazionale per i Servizi Sanitari Regionali-Age.Na.S.), has provided an active evaluation of hospital performance, but the PNE’s indicators regarding perinatal care focus attention on cesarean section only [4]. Also, the 2011–2013 National Health Care Plan underlines the need for development and implementation of certification programs for hospital birth centers, by involving scientific societies, as well as associations of obstetricians and nurses [5, 6].

The quality of care provided to the adult hospitalized Italian population has been scrutinized in the past years by use of adequate indicators [7, 8], whereas only sparse data is available for perinatal care, although an interesting approach suggesting the use of a set of 19 indicators for the performance assessment in the maternity pathway in one region of Italy has thoroughly taken into account also perinatal care indicators [9]. In this context, the primary aim of this study was to assess the quality of perinatal care in a specific geographical area of Italy using the JCAHO indicators, since, compared with PNE indicators, they involve more aspects of perinatal care and include indicators for which there is no standardized monitoring system in Italy, such as breastfeeding or elective delivery [10]. Further aims of the study were to assess the feasibility of these quality indicators in our healthcare setting that could be used to monitor the effects of quality improvement interventions, and to analyze whether maternal, pregnancy care and neonatal characteristics could be associated with the quality of perinatal care hospital performance, measured through JCAHO PC indicators.

## Materials and methods

The study was carried out by retrospectively reviewing medical charts of women over the age of 18 who experienced delivery in Gynecology/obstetrics wards between January 1 and December 31 2016, and those of their newborns hospitalized in the Neonatology or Neonatal Intensive Care Unit (NICU) of a public non-teaching hospital in Catanzaro (Italy).

The medical charts of the women were matched with those of their newborns and reviewed concurrently. Medical charts were selected according to the list of the International Classification of Diseases, 10th Revision, Procedure Coding System (ICD-10-CM/PCS) codes and other diagnosis and procedures codes.

We used the JCAHO perinatal core measure set, which considers the following: Elective deliveries (PC-01); Cesarian section (PC-02) in nulliparous women with a term, singleton baby in a vertex position (NTSV); Antenatal steroids (PC-03); Healthcare-associated bloodstream infections in newborns (PC-04) and Exclusive breast milk feeding (PC-05).

Indicators were calculated according to the methodology specified in the manual for JCAHO measures and summarized in Additional file 1. Whenever the condition described by one of the indicators appeared in the medical record, a score of 1 was assigned if the procedure had been performed consistently with that defined by the indicator, otherwise a score of 0 was attributed.

Since the manual for JCAHO measures does not provide clear target rates, we have used the updated reference goals proposed by NQF and Healthy People 2020, to compare calculated indicators. Regarding the PC-01 indicator, although the optimal rate is considered to be 0% [11], Clark et al. considered that the rate of this indicator will never be and should never be consistently zero [12]. For the PC-02 indicator Healthy People 2020 chose a target of a 23.9% NTSV rate [13]. For the PC-03 and PC-04 indicators, the optimal rate is considered the best performance, that is 100 and 0%, respectively [14, 15]. Finally, for PC-05, a goal of 75% is considered acceptable [16].

Two physicians not involved in patient’ care, but who had been acquainted with the specification manual released at the time of the study design, collected the data and retrieved them on a standardized electronic report form. Maternal, obstetrical and neonatal data were obtained. Specifically, information included socio-demographic and clinical characteristics, obstetric history and pregnancy, delivery, and characteristics of the newborn.

### Statistical analysis

Data analysis was performed through the following steps: 1) for each indicator, frequencies were calculated as the proportion of patients who satisfied the condition of a specific indicator, divided by the total eligible population; 2) then univariate analysis using χ2 test for categorical variables, and Student t-test for independent samples for continuous variables was performed, to explore the association between some PC indicators (elective delivery, cesarian section and exclusive breastfeeding) and several maternal, pregnancy care and neonatal characteristics; 3) furthermore two multivariate logistic regression models were performed to test, after controlling for other variables, the independent association of each of the variables already evaluated at the univariate analysis with the cesarean section in NTSV (model 1) and the adherence to exclusive breast milk feeding measure (model 2). Independent variables for which *p* was 0.25 or less in univariate analysis were included in the multivariate stepwise logistic regression models. The significance level for variables entering the logistic regression models was set at 0.2 for inclusion and at 0.4 for removal from the model. A two-sided *p*-value of 0.05 or less was considered as indicating a statistically significant difference.

In the multivariate logistic regression models, the following independent variables were included if they met the above mentioned criteria: maternal age in years (18–33 = 0; 34–55 = 1), gestational age at delivery in weeks (37–38 = 0; 39–41 = 1), maternal nationality (Italian = 0; other = 1), maternal education (less than high school = 0; high school or higher = 1), prenatal tests (no = 0; yes = 1), Intrauterin Growth Restriction (IUGR) (no = 0; yes = 1), tobacco use during pregnancy (no = 0; yes = 1), pregnancy weight gain in kg (< 10 = 0; ≥ 10 = 1), previous cesarean sections (no = 0; yes = 1), maternal comorbidities (no = 0; yes = 1), rupture of membranes [spontaneous rupture of membranes (SROM) = 0; premature rupture of membranes (PROM) = 1; artificial rupture of membranes (AROM) = 2], place of rupture of membranes (at hospital = 0; at home = 1), amniotic fluid (clear = 0; meconium-stained = 1), amniotic fluid volume (normal = 0; oligohydramnios/polyhydramnios = 1), birth weight in g (< 2500 = 0; 2500–4000 = 1; > 4000 = 2), type of delivery (vaginal = 0; cesarean section = 1). The results of the multivariable models are expressed as odds ratio (OR) with 95% confidence interval (95% CI) and *p* values. Statistical analysis was performed by using STATA software program, version 14 (Stata Corporation. College Station, Tx).

The Ethics Committee of “Mater Domini” Hospital of Catanzaro (Italy) approved the protocol of the study (Prot.E.C.No. 2016/245) in 22 Dec 2016. Considering the nature of the present study, which was based on reviewing medical records of discharged patients, no written consent was needed by the patients.

## Results

A total of 1943 women records were reviewed and, of these, 1172 were eligible for at least one of the PC-Mothers measures (PC-01, 02 and 03). Considering a total of 36 twin-births of which 5 with a single eligible newborn, 1974 newborns’ records were identified and reviewed in order to be included in one of the Newborn PC (PC-04 and 05) subpopulations. The medical records of 297 women were reviewed for the “Elective delivery” indicator, 904 for the “Cesarean section” indicator and 31 for the “Antenatal steroids” indicator. Moreover, 473 newborns’ medical records were reviewed for the “Healthcare-associated bloodstream infections in newborns” indicator, and 1687 for the “Exclusive breast milk feeding” indicator.

Frequency distribution of maternal, pregnancy and prenatal care characteristics according to “Elective delivery”, “Cesarean section” and “Exclusive breast milk feeding” rates are shown in Additional file 2, whereas the scoring of the five PC indicators is reported in Table 1.

**Table 1:** Perinatal Care measure results

JCAHO-PC indicator (^a^)	N (%)	Goal % [Ref.]
PC-01: Elective delivery (297)	82 (27.6)	0 [11]
PC-02: Cesarean section (904)	235 (26)	≤ 23.9 [13]
PC-03: Antenatal steroids (31)	27 (87.1)	100 [14]
PC-04: Healthcare-associated bloodstream infections in newborns (473)	2 (0.4)	0 [15]
PC-05: Exclusive breast milk feeding (1687)	1367 (81)	≥ 75 [16]

^a^In brackets are indicated eligible patients for each indicator

### “Elective delivery” indicator (PC-01)

Elective delivery at > 37 and < 39 weeks of completed gestation (early-term delivery) was performed in more than 25% of the eligible patients.

Of the 82 (27.6%) women who were induced to labor, 20 (24.4%) received medical induction, 56 (68.3%) received medical and surgical induction with AROM, also known as amniorrhexis, and 6 (7.3%) received only amniorrhexis. Elective delivery significantly increased in younger (*χ*^*2*^ *=* 4.28*, p* = 0.039) and nulliparous women (*χ*^*2*^ *=* 43.2*, p* < 0.001). NICU admission did not increase significantly with induction (*χ*^*2*^ *=* 1.15*, p* = 0.284).

### “Cesarean section” indicator (PC-02)

Cesarean section in NTSV was performed in 26% of the eligible population. At univariate analysis, cesarean section was significantly more likely in older women (*χ*^*2*^ *=* 10.85*, p* = 0.001), in those with early-term gestational age (*χ*^*2*^ *=* 7.96, *p* = 0.005), with IUGR (*χ*^*2*^ *=* 6.03*, p* = 0.014), who smoked during pregnancy (*χ*^*2*^ *=* 7.76*, p* = 0.005), in those with comorbidities (*χ*^*2*^ *=* 10.77, *p* = 0.001), in those who underwent amniorrhexis (*χ*^*2*^ *=* 18.13*, p* < 0.001), or had rupture of membranes at hospital (*χ*^*2*^ *=* 5.9, *p* = 0.015), or meconium-stained amniotic fluid (*χ*^*2*^ *=* 31.02, *p* < 0.001), and those who delivered a newborn with > 4000 g birth weight (*χ*^*2*^ *=* 31.6*, p* < 0.001).

Results of the multivariate stepwise logistic regression analysis confirmed those of the univariate analysis, except for maternal age, IUGR and maternal comorbidities that were no more significantly associated with cesarean section in NTSV; location of membrane rupture was removed from the model (Model 1 in Table 2).

**Table 2:** Multiple logistic regression analysis results examining perinatal care quality measures according to several explanatory variables

Variable	OR	SE	95% CI	*p*	OR	SE	95% CI	*p*
	*Model 1. Cesarean section indicator*				*Model 2. Exclusive breast milk feeding indicator*			
	*Log likelihood = − 325.29, χ*^*2*^ *= 69.07 (10 df), p < 0.00001, No. of obs = 622*				*Log likelihood = −498.52, χ*^*2*^ *= 79.69 (10 df), p < 0.00001, No. of obs = 1103*			
Maternal age (years)								
18–33	1.00^a^				1.00^a^			
34–55	1.42	0.30	0.93–2.17	0.100	0.60	0.10	0.43–0.83	0.002
Gestational age at delivery (weeks)								
37–38	1.00^a^				1.00^a^			
39–41	0.59	0.13	0.38–0.91	0.019	1.60	0.29	1.12–2.29	0.010
Maternal nationality								
Italian	1.00^a^				NA^b^			
Other	0.50	0.21	0.22–1.13	0.097	NA^b^			
Prenatal tests^c^								
No	NA^b^				1.00^a^			
Yes	NA^b^				1.87	0.33	1.32–2.65	< 0.001
IUGR^d^								
No	1.00^a^				1.00^a^			
Yes	2.00	0.85	0.87–4.63	0.103	0.46	0.24	0.16–1.29	0.140
Tobacco use during pregnancy								
No	1.00^a^				1.00^a^			
Yes	2.42	0.86	1.20–4.87	0.013	0.61	0.20	0.31–1.18	0.141
Pregnancy weight gain (Kg)								
< 10	NA^b^				1.00^a^			
> 10	NA^b^				1.26	0.20	0.92 - 1.74	0.152
Maternal comorbidities^e^								
No	1.00^a^				NA^b^			
Yes	1.51	0.36	0.94–2.43	0.086	NA^b^			
Previous cesarean sections								
No	NA^b^				1.00^a^			
Yes	NA^b^				1.83	0.49	1.08–3.11	0.025
Rupture of membranes^f^								
SROM	1.47	0.36	0.90–2.38	0.123	1.00^a^			
PROM	1.00^a^				Backward elimination			
AROM	2.01	0.46	1.28–3.15	0.002	0.70	0.12	0.51–0.97	0.035
Amniotic fluid								
Clear	1.00^a^				NA^b^			
Meconium-stained	3.08	0.77	1.89–5.01	< 0.001	NA^b^			
Amniotic fluid volume								
Normal	NA^b^				1.00^a^			
Oligohydramnios / polyhydramnios	NA^b^				0.56	0.22	0.26–1.19	0.134
Birth weight (g)								
< 2500	Backward elimination				0.26	0.10	0.12–0.56	0.001
2500–4000	1.00^a^				1.00^a^			
> 4000	6.36	3.45	2.20–18.40	0.001	2.57	1.63	0.74–8.89	0.135
Type of delivery								
Vaginal	NA^b^				1.00^a^			
Cesarean section	NA^b^				0.39	0.78	0.26 - 0.57	< 0.001

^a^Reference category

^b^Not Applicable

^c^Amniocentesis, funicolocentesis, fetoscopy, Chorionic Villus Sampling (CVS), Non Invasive Prenatal Testing (NIPT)

^d^Intrauterin Growth Restriction

^e^Hypertension and diabetes

^f^*SROM* spontaneous rupture of membranes, *PROM* premature rupture of membranes, *AROM* artificial rupture of membranes (amniorrhexis)

Among the eligible newborns (904), we found that NICU admission (7%) and hypoxia (3%) increased significantly with cesarean section (*χ*^*2*^ *=* 10.01, *p* = 0.002 and *χ*^*2*^ *=* 7.1, *p* = 0.008, respectively).

### “Antenatal steroids” indicator (PC-03)

Only 31 women delivered at > 24 and < 34 weeks of gestation and were considered eligible for antenatal steroids administration. Of these, 87.1% received at least one dose of antenatal steroids before delivering preterm newborns; specifically 9 (29%) did not receive any therapy, 6 (19.3%) received one dose, 14 (45.2%) received 2 doses, and 2 (6.5%) received more than 2 doses. For all women each dose consisted of 12 mg intramuscular betamethasone.

All 36 newborns, whose mothers were eligible for antenatal steroids administration, required NICU admission. Respiratory distress syndrome (RDS) occurred in about 60% of these newborns regardless of having been exposed to steroids, whereas mechanical ventilation was required by 60% of newborns who were not exposed to steroids compared to 48.4% of those whose mothers received steroids, although no significant differences were revealed by univariate analysis between the two groups.

### “Healthcare-associated bloodstream infections in newborns” indicator (PC-04)

Of the 473 eligible newborns, only 2 (0.4%) developed a microbiologically confirmed Healthcare-associated bloodstream infection caused by Coagulase-negative staphylococci. The mean birth weight was 2834 g (range 620 to 4770 g), 42.3% of newborns needed NICU admission, and the most common primary diagnoses were prematurity, small for gestational age (SGA), and RDS. Neonatal comorbidities, mostly RDS, occurred more frequently in newborns with a gestation period of ≥ 30 weeks (77.5%).

### “Exclusive breast milk feeding” indicator (PC-05)

Exclusive breast milk feeding at hospital discharge was performed in more than 80% of the eligible newborns. It significantly increased in older women (*χ*^*2*^ *=* 19.67, *p* < 0.001), in those with late-term gestational age (*χ*^*2*^ *=* 26.05, *p* < 0.001), those who underwent prenatal tests (*χ*^*2*^ *=* 4.01, *p* = 0.045), without IUGR (*χ*^*2*^ *=* 0.01, *p* < 0.001), with pregnancy weight gain > 10 kg (*χ*^*2*^ *=* 8.24, *p* = 0.004), without previous cesarean sections (*χ*^*2*^ *=* 9.39, *p* < 0.001), without comorbidities (*χ*^*2*^ *=* 4.67, *p* = 0.031), with SROM (*χ*^*2*^ *=* 8.52, *p* = 0.014), with normal amniotic fluid volume (*χ*^*2*^ *=* 6.26, *p* = 0.044), with newborn birth weight between 2500 and 4000 g (*χ*^*2*^ *=* 48.06, *p* < 0.001), and with vaginal delivery (*χ*^*2*^ *=* 52, *p* < 0.001).

Multivariate stepwise logistic regression analysis results underlined those of the univariate analysis, except for IUGR, pregnancy weight gain and amniotic fluid volume that were no longer significantly associated with adherence to exclusive breast milk feeding; moreover, maternal education and maternal comorbidities were removed from the model (Model 2 in Table 2).

## Discussion

Perinatal care quality and safety is a complex process aimed at achieving maximum health potential for the fetus, the newborn and the mother, and, similarly, evaluating quality of perinatal care is complex because it involves different populations. This is the first study performed in Italy that has evaluated the quality of perinatal care by using all the five JCAHO PC indicators. The application of this feasible set of indicators allowed us to measure several aspects of perinatal care for which there is no standardized monitoring system in Italy, as well as the factors associated with an eventual suboptimal performance. Overall, the results pointed out that the quality of perinatal hospital care, measured through the JCAHO PC indicators, is indicator dependent, with exclusive breastfeeding performing well, whereas for most indicators there is room for improvement.

The most critical result of our study pertains to the elective/early-term delivery, that has been performed in 27.6% of eligible women, far from the goal (0%) set by Clark et al., that strongly confirmed the commitment to the elimination of early term elective delivery [12]. This result is concerning, since this indicator has been reported to be one of the most important performance measures due to its impact on clinical practice, on healthcare costs, and on patients’ morbidity [17]. Albeit, Salemi et al. in a recent cohort study, highlighted that no excess risk of respiratory morbidities, neonatal sepsis, and NICU admission was associated with the elective induction of delivery at 37–38 weeks of gestation with respect to infants expectantly managed and delivered at 39–40 weeks. Only the early cesarean section significantly supports 13 to 66% increase of several adverse outcomes occurrence in neonates, when compared with the full-term group [18].

In Italy, the practice of induction of labor and elective cesarean section are investigated separately. The Europeristat project has detected that inductions were performed in 15.9% of the total births in Italy in 2010, but data do not allow a separate evaluation of the induction performed in early term deliveries [19]. Therefore, there is a need to standardize definitions and evaluation methods of elective early term deliveries, in order to improve the validity of comparisons among countries. Moreover, as reported by Clark et al. [17], the calculation of this indicator is prone to errors particularly related to the selection of clinical indications for inclusion/exclusion of elective delivery.

The result of the PC-02 indicator is in line with the Italian national figure of cesarean section rate that is recorded as one of the highest among European countries [20]. Several reasons may be related to the high cesarean rate found in our study: first of all, the cesarean section high rate may be associated with the changed attitude of the physicians aimed at reducing exposure to malpractice litigation [21]. Indeed, in our results cesarean delivery was significantly more frequent in pregnancy with AROM, meconium-stained amniotic fluid and newborns with > 4000 g birth weight, that are not medical indications for cesarean section, suggesting therefore that choice to perform cesarean section was related to a cautious approach of physicians to delivery. Moreover, women’s choice of cesarean delivery is increasing since it is perceived as an effective procedure to avoid pain and the other disadvantages associated with vaginal delivery [22, 23]. Finally, cesarean section has been reported to be more frequently performed in the private healthcare sector than in the public one [24–26] and one of the main reasons for this is that cesarean sections receive higher reimbursement than normal vaginal births, regardless of the risks to women [27] and private healthcare facilities are commonly involved in deliveries in Southern Italy [24, 28, 29].

It is well known that cesarean sections should be discouraged because they create serious complications for mothers such as infections [30], obstetric haemorrhage [31], uterine rupture, stillbirth and pre-term birth [32]. Also, there is emerging evidence that children born by cesarean section have an increased risk of altered immune development, allergy and asthma, and reduced intestinal gut microbiome diversity [32]. Moreover, as our results have shown and according to previous studies, cesarean sections have a negative effect on the exclusive breast milk feeding, probably because they limit the practice of rooming-in, so delaying the mother-child interaction [33, 34]. Thus, cesarean section should not be considered an alternative to vaginal delivery, and should be viewed with caution. Indeed, among the measures taken to discourage unnecessary cesarean sections, several countries have also narrowed the gap in hospital payment between a cesarean section and a vaginal birth [35].

The exclusive breast milk feeding indicator achieved the best performance (81%) in our study. In particular, our result is higher than the set goal (75%) [16], and higher than the value found in 2015 in Italy (77%) [36]. However, it is well known that in Italy there is a tendency to wean children from breastfeeding at an early age, on average at 4 months [29] although, as previously reported, the start of exclusive breast milk feeding during hospitalization positively influences the continuation of this practice in the following months [33, 34, 37].

Health professionals’ approaches to breastfeeding during antenatal care are crucial to promote exclusive breast milk feeding. Our results highlight that exclusive breast milk feeding significantly increased in women who underwent prenatal tests; probably this practice contributed in encouraging increasing educational activities in physician-patient relationship also regarding breastfeeding. Also, avoiding in-hospital formula supplementation appears to be a key step for breastfeeding success, together with the appropriate implementation of the other Baby Friendly Hospital Initiative (BFHI) steps [38]. As highlighted by recent meta-analyses, the BFHI approach by steps requiring implementation at the maternity ward, followed by home and family support through counselling, appears to be crucial for breastfeeding success in expectant and/or nursing mothers [39, 40]. The significantly lower adherence to PC-05 of newborns with a birth weight < 2500 g as well as in women with previous cesarean section, AROM and, as previous mentioned, with cesarean delivery, are important concerns of our results. These findings suggest the involvement of several non-clinical factors that would seem to be attributable to the overly cautious attitude of the physician concerning patients’ management, suggesting the need for improvement in the training of healthcare professionals.

Antenatal steroids are intended to reduce the burden of prematurity-related illness (respiratory distress, intraventricular haemorrhage, necrotizing enterocolitis, and patent ductus arteriosus) in preterm newborns. The prevalence of early preterm infants revealed by our study (1.8%) is in line with previous data [41], nevertheless there are concerns in international comparisons for this quality measure due to the differences in registration criteria and definitions across countries [42]. This indicator was satisfied in only 87.1% of the eligible patients, highlighting a suboptimal process of care that might have led to increased morbidity or mortality. Indeed, in our study, when the PC-03 indicator was not satisfied, newborns more frequently experienced mechanical ventilation compared with newborns whose mother received antenatal steroids; however, these results are to be interpreted with caution, because of the limited number of included patients.

As underlined in a recently published meta-analysis, there is continuing uncertainty about the most appropriate method to calculate the healthcare-associated bloodstream infections burden in NICUs. Cumulative incidence of these severe complications is reported to be variable from 2.9 to 22.8% [43]. Only two healthcare-associated bloodstream infections occurred during 2016 (0.4%) and this result allows us to interpret compliance with the PC-04 indicator as to be satisfied, although also in this case a cautious interpretation of the results is needed regarding the above mentioned concerns in the calculation of this indicator.

Although the application of JCAHO PC quality indicators was feasible and intuitive, results of this study should be evaluated in light of potential limitations, considering the fragmentary availability of required data. First, the poor comparability among multiple classification systems is the most substantial barrier that we met. Gilbert et al. suggested that improvement of performance’s quality depends on the improvement of the accuracy of data recording and its transparency [1]. Second, patients were recruited from a hospital located in Southern Italy, and may not be representative of the entire country. Third, most of the previous studies were conducted on large numbers of hospitals and therefore were based on aggregated data. Instead, by focusing on one hospital, our results were derived from a smaller number of patients, but detailed information was gathered from each of them.

In conclusion, our findings revealed significant deficiencies in the adherence to recommended processes of perinatal care and, consistently with previous studies conducted by some of us to estimate the adherence to evidence-based processes of care in several settings [7, 44, 45], suggest that it is essential to increase efforts to implement evaluation processes that reflect the healthcare quality based on current evidence and related practice guidelines. The application of the JCAHO PC indicators has demonstrated to be feasible, intuitive and useful to measure perinatal hospital performance, and, although the poor comparability among multiple available quality measures represents a barrier, these performance metrics can be reliably used within an institution, thus enabling comparisons of performance over time, particularly after the implementation of quality improvement interventions.

## Additional files

Additional file 1: Quality indicators for perinatal care improvement. Methodology of indicators calculated specified in the manual for JCAHO measures. (DOCX 17 kb)

Additional file 2: Overall adherence to JCHAO indicators and according to several maternal, pregnancy, prenatal care and neonatal characteristics. Frequency distribution of maternal, pregnancy and prenatal care characteristics according to “Elective delivery”, “Cesarean section” and “Exclusive breast milk feeding” rates. (DOCX 34 kb)

## Abbreviations

Age.Na.S: Agenzia Nazionale per i servizi sanitari Regionali (National Agency for Regional Health Services)
AROM: Artificial rupture of membranes
CI: Confidence interval
IUGR: Intrauterin growth restriction
JCAHO: Joint commission on accreditation of healthcare organizations
NICU: Neonatal intensive care unit
NQF: National quality forum
NTSV: Nulliparous women with a Term, Singleton baby in a Vertex position
OR: Odds ratio
PC: Perinatal care
PNE: Piano Nazionale Esiti (Outcomes National Plan)
PROM: Premature rupture of membranes
RDS: Respiratory distress syndrome
SGA: Small for gestational age
SROM: Spontaneous rupture of membranes
