# Supplementary material for: Quality in perinatal care: applying performance measurement using joint commission on accreditation of healthcare organizations indicators in Italy
Source: BMC Med Res Methodol. 2019 Apr 24;19:83. doi: 10.1186/s12874-019-0722-z (PMC6480744; doi:10.1186/s12874-019-0722-z)
Supplement: Supplementary file 1 — Quality indicators for perinatal care improvement. Methodology of indicators calculated specified in the manual for JCAHO measures. (DOCX 17 kb) [file 12874_2019_722_MOESM1_ESM.docx]

**Additional file 1. Quality indicators for perinatal care improvement**

| **Measure** | **Denominator** | **Numerator** | **Patients excluded** | **Goal** |
| --- | --- | --- | --- | --- |
| **Perinatal Care (PC)** |  |  |  |  |
| Elective delivery  (PC-01) | Patients who delivered at >37 and <39 weeks of completed gestation | Patients from the denominator:   - who received medical and/or surgical induction of labor - with cesarean section without history of prior uterine surgery and not in labor | Patients:   - who had less than 8 or greater than 64 years old - with length of stay greater than 120 days - with medical and obstetric ICD-9-CM diagnoses codes that possibly justifying delivery prior to 39 weeks of gestation | 0%  [11] |
| Cesarean section  (PC-02) | Nulliparous patients with a term, singleton baby in a vertex presentation (NTSV) | Patients from the denominator with cesarean delivery | Patients:   - who had less than 8 or greater than 64 years old - with length of stay greater than 120 days - with less than 37 weeks of gestation - with ICD-9-CM diagnosis codes representing contraindications to vaginal deliveries | ≤23.9%  [13] |
| Antenatal steroids  (PC-03) | Patients who delivered preterm newborns at > 24 and <34 weeks of completed gestation | Patients from the denominator who received antenatal steroids before delivering preterm newborns | Patients:   - who had less than 8 or greater than 64 years old - with length of stay greater than 120 days - with presence of documented reason for not initiating antenatal steroids - with intrauterine death | 100%  [14] |
| Healthcare-associated bloodstream infections in newborns  (PC-04) | Live-born newborns:   - with ICD-9-CM other diagnosis codes for birth weight between 500 and 1499 g, or birth weight between 500 and 1499 g - with ICD-9-CM other diagnosis codes for birth weight greater than 1499 g, or with a birth weight greater than 1499 g, if the infant: - experienced death - major surgery - mechanical ventilation - transfer in from another acute care hospital or healthcare setting within 2 days of birth | Total number of newborns from the denominator:   - with ICD-9-CM other diagnosis codes for staphylococcal and gram-negative septicemias or bacteremias - with ICD-9-CM other diagnosis codes for sepsis | Newborns:   - with length of stay less than 2 days - with ICD-9-CM principal diagnosis codes for septicemias or bacteremias - with ICD-9-CM other diagnosis codes for septicemias or bacteremias or with ICD-9-CM for newborn septicemia or bacteremia diagnosis codes with a bloodstream infection present on admission - with ICD-9-CM other diagnosis codes for birth weight less than 500 g, or birth weight less than 500 g | 0%  [15] |
| Exclusive breast milk feeding  (PC-05) | Single live-born newborn delivered at >37 and <42 weeks of completed gestation with ICD-9-CM principal diagnosis codes for single live-born newborn discharged alive from the hospital | Newborns who were fed breastmilk only at hospital discharge | Newborns:   - admitted to the NICU - who received parental infusion - with ICD-9-CM other diagnosis codes for galactosemia - who were transferred to another hospital - who experienced death - with documented reason for not exclusively feeding breastmilk - with length of stay greater than 120 days | ≥75%  [16] |
