# Supplementary material for: Quality in perinatal care: applying performance measurement using joint commission on accreditation of healthcare organizations indicators in Italy
Source: BMC Med Res Methodol. 2019 Apr 24;19:83. doi: 10.1186/s12874-019-0722-z (PMC6480744; doi:10.1186/s12874-019-0722-z)
Supplement: Supplementary file 2 — Overall adherence to JCHAO indicators and according to several maternal, pregnancy, prenatal care and neonatal characteristics. Frequency distribution of maternal, pregnancy and prenatal care characteristics according to “Elective delivery”, “Cesarean section” and “Exclusive breast milk feeding” rates. (DOCX 34 kb) [file 12874_2019_722_MOESM2_ESM.docx]

**Additional file 2. Overall adherence to JCAHO indicators and according to several maternal, pregnancy, prenatal care and neonatal characteristics**

| **Characteristics** | **Elective delivery** | | | | | **Cesarean section** | | | | **Exclusive breast milk feeding** | | |
| --- | --- | --- | --- | --- | --- | --- | --- | --- | --- | --- | --- | --- |
|  | **Eligible patients*** | | **Eligible patients who met the indicator*** | |  | **Eligible patients*** | | **Eligible patients who met the indicator*** |  | **Eligible patients*** | **Eligible patients who met the indicator*** |  |
|  | **n (%)** | | **n (%)** | |  | **n (%)** | | **n (%)** |  | **n (%)** | **n (%)** |  |
| **Total** | 297 | | 82 (27.6) | |  | 904 | | 235 (26) |  | 1687 | 1367 (81) |  |
| **Maternal age (years)** |  | | | |  |  | | |  |  | |  |
| 18-33 | 145 (48.8) | | 48 (33.1) | | ***χ^2^=4.28, 1df***  ***p=0.039*** | 659 (72.9) | | 152 (23.1) | ***χ^2^=10.85, 1df***  ***p=0.001*** | 997 (59.1) | 843 (84.5) | ***χ^2^=19.67, 1df***  ***p<0.001*** |
| 34-55 | 152 (51.2) | | 34 (22.4) | |  | 245 (27.1) | | 83 (33.9) |  | 690 (40.9) | 524 (75.9) |  |
| **Gestational age at delivery (weeks)** |  | | | |  |  | | |  |  | |  |
| 37-38 | NA^ψ^ | | NA^ψ^ | | NA^ψ^ | 230 (25.4) | | 76 (33) | ***χ^2^=7.96, 1df***  ***p=0.005*** | 570 (33.8) | 423 (74.2) | ***χ^2^=26.05, 1df***  ***p<0.001*** |
| 39-41 | NA^ψ^ | | NA^ψ^ | |  | 674 (74.6) | | 159 (23.4) |  | 1117 (66.2) | 944 (84.5) |  |
| **Maternal nationality** |  | | | |  |  | | |  |  | |  |
| Italian | 278 (93.6) | | 74 (26.6) | | χ^2^=2.13, 1df  p=0.144 | 824 (91.1) | | 221 (26.8) | χ^2^=3.29, 1df  p=0.070 | 1537 (91.1) | 1243 (80.9) | χ^2^=0.28, 1df  p=0.593 |
| Other | 19 (6.4) | | 8 (42.1) | |  | 80 (8.8) | | 14 (17.5) |  | 150 (8.9) | 124 (82.7) |  |
| **Marital status** |  | | | |  |  | | |  |  | |  |
| Other | 72 (24.2) | | 23 (31.9) | | χ^2^=0.89, 1df  p=0.345 | 257 (28.4) | | 66 (25.7) | χ^2^=0.02, 1df  p=0.892 | 398 (23.6) | 322 (80.9) | χ^2^=0.01, 1df  p=0.941 |
| Married | 225 (75.8) | | 59 (26.2) | |  | 647 (71.6) | | 169 (26.1) |  | 1289 (76.4) | 1045 (81.1) |  |
| **Maternal education** |  | | | |  |  | | |  |  | |  |
| Less than HS^¥^ | 66 (23.3) | | 13 (19.7) | | χ^2^=4.85, 2df  p=0.088 | 142 (16.5) | | 38 (26.8) | χ^2^=0.599, 2df  p=0.741 | 334 (20.7) | 258 (77.2) | χ^2^=3.91, 2df  p=0.141 |
| HS^¥^ | 131 (46.3) | | 44 (33.6) | |  | 404 (47) | | 109 (27) |  | 738 (45.7) | 608 (82.4) |  |
| Higher than HS^¥^ | 86 (30.4) | | 21 (24.4) | |  | 314 (36.5) | | 77 (24.5) |  | 541 (33.5) | 437 (80.8) |  |
| **Prenatal tests^⁂^** |  | | | |  |  | | |  |  | |  |
| No | 159 (53.5) | 41 (25.8) | | | χ^2^=0.57, 1df  p=0.451 | 491 (54.3) | 122 (24.8) | | χ^2^=0.23, 1df  p=0.629 | 917 (54.4) | 727 (79.3) | ***χ^2^=4.01, 1df***  ***p=0.045*** |
| Yes | 138 (46.5) | 41 (29.7) | | |  | 413 (45.7) | 113 (27.4) | |  | 770 (45.6) | 640 (83.1) |  |
| **IUGR**^†^ |  | | | |  |  | | |  |  | |  |
| No | NA^ψ^ | NA^ψ^ | | | NA^ψ^ | 870 (96.2) | 220 (25.3) | | ***χ^2^=6.03, 1df***  ***p=0.014*** | 1660 (98.4) | 1355 (81.6) | ***χ^2^=0.01, 1df***  ***p<0.001*** |
| Yes | NA^ψ^ | NA^ψ^ | | |  | 34 (3.8) | 15 (44.1) | |  | 27 (1.6) | 12 (44.4) |  |
| **Tobacco use during pregnancy** |  | | | |  |  | | |  |  | |  |
| No | 215 (93.9) | 57 (26.5) | | | Fisher’s exact p=0.200 | 650 (94.1) | 171 (26.3) | | ***χ^2^=7.76, 1df***  ***p=0.005*** | 1187 (95.1) | 956 (80.5) | χ^2^=2.56, 1df  p=0.109 |
| Yes | 14 (6.1) | 1 (7.1) | | |  | 41 (5.9) | 19 (46.4) | |  | 61 (4.9) | 44 (72.1) |  |
| **Pregnancy weight gain (Kg)** |  | | | |  |  | | |  |  | |  |
| <10 | 144 (48.5) | 34 (23.6) | | | χ^2^=2.23, 1df  p=0.135 | 363 (40.1) | 100 (27.5) | | χ^2^=0.46, 1df  p=0.383 | 754 (44.7) | 588 (77.9) | ***χ^2^=8.24, 1df***  ***p=0.004*** |
| >10 | 153 (51.5) | 48 (31.4) | | |  | 541 (59.9) | 135 (24.9) | |  | 933 (55.3) | 779 (83.5) |  |
| **Threatened abortion and/or preterm labor** |  | | | |  |  | | |  |  | |  |
| No | 191 (64.3) | 48 (25.1) | | | χ^2^=1.64, 1df  p=0.200 | 649 (71.8) | 172 (26.5) | | χ^2^=0.31, 1df  p=0.579 | 1212 (71.8) | 984 (81.2) | χ^2^=0.07, 1df  p=0.793 |
| Yes | 106 (35.7) | 34 (32.1) | | |  | 255 (28.2) | 63 (24.7) | |  | 475 (28.2) | 383 (80.6) |  |
| **Parity** |  | | | |  |  | | |  |  | |  |
| Nulliparous | 84 (28.3) | 46 (54.8) | | | ***χ^2^=43.2, 1df***  ***p<0.001*** | NA^ψ^ | NA^ψ^ | | NA^ψ^ | 877 (52) | 704 (80.3) | χ^2^=0.68, 1df  p=0.409 |
| Multiparous | 213 (71.7) | 36 (16.9) | | |  | NA^ψ^ | NA^ψ^ | |  | 810 (48) | 663 (81.8) |  |
| **Previous cesarean sections** |  | | | |  |  | | |  |  | |  |
| No | 164 (55.2) | NA^ψ^ | | | NA^ψ^ | NA^ψ^ | NA^ψ^ | | NA^ψ^ | 1455 (86.2) | 1196 (82.2) | ***χ^2^=9.39, 1df***  ***p<0.001*** |
| Yes | 133 (44.8) | NA^ψ^ | | |  | NA^ψ^ | NA^ψ^ | |  | 232 (13.7) | 171 (73.7) |  |
| **Maternal comorbidities^Δ^** |  | | | |  |  | | |  |  | |  |
| No | NA^ψ^ | NA^ψ^ | | | NA^ψ^ | 751 (83.1) | 179 (23.8) | | ***χ^2^=10.77, 1df p=0.001*** | 1436 (85.1) | 1176 (81.9) | ***χ^2^=4.67, 1df***  ***p=0.031*** |
| Yes | NA^ψ^ | NA^ψ^ | | |  | 153 (16.9) | 56 (36.6) | |  | 251 (14.9) | 191 (76.1) |  |
| **Rupture of membranes°** |  | | | |  |  | | |  |  | |  |
| SROM | 225 (78.4) | NA^ψ^ | | | NA^ψ^ | 218 (25.4) | 63 (28.9) | | ***χ^2^=18.13, 2df p<0.001*** | 548 (33.7) | 458 (83.6) | ***χ^2^=8.52, 2df***  ***p=0.014*** |
| PROM | NA^ψ^ | NA^ψ^ | | |  | 380 (44.2) | 72 (18.9) | |  | 522 (32.1) | 433 (82.9) |  |
| AROM | 62 (21.6) | NA^ψ^ | | |  | 261 (30.4) | 87 (33.3) | |  | 556 (34.2) | 430 (77.3) |  |
| **Place of membranes’ rupture** |  | | | |  |  | | |  |  | |  |
| At hospital | 285 (97.9) | 78 (27.4) | | | Fisher’s exact p=0.668 | 748 (85.1) | 207 (27.7) | | ***χ^2^=5.90, 1df p=0.015*** | 1453 (88.5) | 1173 (80.7) | χ^2^=0.59, 1df  p=0.441 |
| At home | 6 (2.1) | 2 (33.3) | | |  | 131 (14.9) | 23 (17.6) | |  | 189 (11.5) | 157 (83.1) |  |
| **Amniotic fluid** |  | | | |  |  | | |  |  | |  |
| Clear | NA^ψ^ | NA^ψ^ | | | NA^ψ^ | 739 (84.5) | 161 (21.8) | | ***χ^2^=31.02, 1df p<0.001*** | 1454 (88.5) | 1183 (81.4) | χ^2^=0.695, 1df  p=0.404 |
| Meconium-stained | NA^ψ^ | NA^ψ^ | | |  | 135 (15.4) | 60 (44.4) | |  | 189 (11.5) | 149 (78.8) |  |
| **Amniotic fluid volume** |  |  | |  | |  |  | |  |  |  |  |
| Normal | NA^ψ^ | NA^ψ^ | | NA^ψ^ | | 778 (94.8) | 195 (25.1) | | χ^2^=2.50, 2df p=0.286 | 1524 (96.1) | 1254 (82.3) | ***χ^2^=6.26, 2df***  ***p=0.044*** |
| **Polyhydramnios** | NA^ψ^ | NA^ψ^ | |  |  | 11 (1.3) | 5 (45.4) | |  | 19 (1.2) | 12 (63.2) |  |
| **Oligohydramnios** | NA^ψ^ | NA^ψ^ | |  |  | 32 (3.9) | 9 (28.1) | |  | 43 (2.7) | 32 (74.4) |  |
| **Birth weight (g)** |  |  | |  | |  |  | |  |  |  |  |
| <2500 | 14 (4.7) | 4 (28.6) | | Fisher’s exact p=0.826 | | 44 (4.9) | 16 (36.4) | | ***χ^2^=31.60, 2df p<0.001*** | 49 (2.9) | 21 (42.9) | ***χ^2^=48.06, 2df p<0.001*** |
| 2500-4000 | 278 (93.6) | 76 (27.3) | |  |  | 835 (92.4) | 201 (24.1) | |  | 1589 (94.2) | 1307 (82.3) |  |
| >4000 | 5 (1.7) | 2 (40) | |  |  | 25 (2.8) | 18 (72) | |  | 49 (2.9) | 39 (79.6) |  |
| **Type of delivery** |  |  | |  | |  |  | |  |  |  |  |
| Vaginal | NA^ψ^ | NA^ψ^ | | NA^ψ^ | | NA^ψ^ | NA^ψ^ | | NA^ψ^ | 1159 (68.7) | 993 (85.7) | ***χ^2^=52.00, 1df p<0.001*** |
| Cesarean section | NA^ψ^ | NA^ψ^ | |  |  | NA^ψ^ | NA^ψ^ | |  | 528 (31.3) | 374 (70.8) |  |

*Total may not always sum to “n” because of missing data

^ψ^Not Applicable

^¥^High School

**^⁂^**Amniocentesis, funicolocentesis, fetoscopy, Chorionic Villus Sampling (CVS), Non Invasive Prenatal Testing (NIPT)

^†^Intrauterin Growth Restriction

^Δ^Hypertension and diabetes

°SROM: spontaneous rupture of membranes; PROM: premature rupture of membranes; AROM: artificial rupture of membranes (amniorrhexis)
